# Nucleotide diversity and linkage disequilibrium in 11 expressed resistance candidate genes in *Lolium perenne*

**DOI:** 10.1186/1471-2229-7-43

**Published:** 2007-08-04

**Authors:** Yongzhong Xing, Uschi Frei, Britt Schejbel, Torben Asp, Thomas Lübberstedt

**Affiliations:** 1 University of Århus, Faculty of Agricultural Sciences, Department of Genetics and Biotechnology, Research Centre Flakkebjerg, Slagelse DK-4200, Denmark; 2 National Key Lab of Crop Genetic Improvement, Huazhong Agricultural University, Wuhan 430070, China

## Abstract

**Background:**

Association analysis is an alternative way for QTL mapping in ryegrass. So far, knowledge on nucleotide diversity and linkage disequilibrium in ryegrass is lacking, which is essential for the efficiency of association analyses.

**Results:**

11 expressed disease resistance candidate (R) genes including 6 nucleotide binding site and leucine rich repeat (NBS-LRR) like genes and 5 non-NBS-LRR genes were analyzed for nucleotide diversity. For each of the genes about 1 kb genomic fragments were isolated from 20 heterozygous genotypes in ryegrass. The number of haplotypes per gene ranged from 9 to 27. On average, one single nucleotide polymorphism (SNP) was present per 33 bp between two randomly sampled sequences for the 11 genes. NBS-LRR like gene fragments showed a high degree of nucleotide diversity, with one SNP every 22 bp between two randomly sampled sequences. NBS-LRR like gene fragments showed very high non-synonymous mutation rates, leading to altered amino acid sequences. Particularly LRR regions showed very high diversity with on average one SNP every 10 bp between two sequences. In contrast, non-NBS LRR resistance candidate genes showed a lower degree of nucleotide diversity, with one SNP every 112 bp. 78% of haplotypes occurred at low frequency (<5%) within the collection of 20 genotypes. Low intragenic LD was detected for most R genes, and rapid LD decay within 500 bp was detected.

**Conclusion:**

Substantial LD decay was found within a distance of 500 bp for most resistance candidate genes in this study. Hence, LD based association analysis is feasible and promising for QTL fine mapping of resistance traits in ryegrass.

## Background

Perennial ryegrass is a diploid out-breeding species with a strong self-incompatibility system. Major agronomic traits for this species such as forage quality are quantitatively inherited [1]. Molecular (DNA) markers have recently become available and employed to study different characters including vernalisation response [2], forage quality [3], and disease resistances [4,5].

QTL mapping has been demonstrated as a successful method to dissect the genetic bases of complex traits in several important crops since the 1990s. However, due to its self-incompatibility, populations of doubled haploid lines (DHs) or recombinant inbred lines (RILs) favorable for QTL mapping in other crops are difficult to develop in ryegrass. Therefore, populations for QTL mapping in ryegrass are mainly pseudo-F_2 _populations derived from heterozygous parents [2,5]. Thus, each polymorphic locus might segregate for more than two and up to four alleles. Consequently, a large population size is required for reliable and high resolution QTL detection. Association studies based on linkage disequilibrium (LD) mapping could be an alternative and more efficient way for QTL/gene tagging in ryegrass. The high degree of genetic variation between and within ryegrass populations might be beneficial for identification of both genes and polymorphisms affecting quantitative inherited characters for development of informative "functional markers" [6].

LD is the non-independence of alleles, based on non-random allelic association at different loci, and is proportional to the recombination fraction. LD and association studies have been performed in plants recently (reviewed by Gupta et al. [7].). Many factors affect LD including mating patterns, genetic drift, population admixture, selection, and mutations. Several measures were used for LD estimation [7]. Standardized disequilibrium coefficients (D') [8] and squared allele-frequency correlations (r^2^) [9] for pairs of loci are the preferred measures of LD. D' is only affected by recombination rate, whereas r^2 ^is also affected by differences in the allele frequency at the two sites.

In coalescent simulations, high levels of selfing greatly increase levels of LD [10]. In out-crossing species, LD will often decay within 500 bp, but for highly autogamous species LD may exceed 10 kb. However, different gene regions exhibit very different structures of LD even within the same species. In maize, rapid LD decay was observed within 1500 bp at *d3*, *id1*, *tb1*, and *sh1*, whereas at *su1 *LD extended to 7 kb [11]. High levels of LD were observed among single nucleotide polymorphisms (SNPs) up to 600 kb in a region surrounding the *Y1 *gene [12] and a 500 kb region around the *sdh1 *gene [13]. The structure of LD can be locus specific due to varied recombination and mutation rates or natural selection pressure, but is also highly population-specific [14]. Generally low levels of LD are expected in ryegrass due to its out-crossing and heterozygous nature. The only report on the structure of LD in ryegrass [15] has been performed at a genome-wide scale using genetic markers. In ryegrass, LD has so far not been analyzed for candidate genes.

DNA sequence mutations, especially SNPs, can either directly determine a phenotype or be closely associated with a phenotype as a result of linkage disequilibrium [14]. Moreover, SNPs have been widely surveyed in several species to address evolutionary questions [16-18]. Resistance genes are very abundant in plant genomes and the majority belongs to clustered gene families. So far, sequence diversity of resistance genes has mainly been studied in Arabidopsis [19]. In this study, we sequenced about 1 kb regions of 11 expressed disease resistance candidate genes from 20 heterozygous genotypes (Lolium Test Set, LTS) employed in the EU project GRASP [20]. The goal in GRASP was to perform SNP assays for candidate gene allele tracing in selection experiments. The objectives of this study were to (1) identify SNPs for allele tracing in GRASP within about 1 kb fragments of expressed resistance candidate genes, (2) compare the nucleotide diversity within and between different resistance candidate genes, (3) determine the extent and structure of LD within these genes, and (4) discuss the prospects of candidate-gene based association mapping in ryegrass.

## Results

### Haplotypes and homozygosity

**Figure 1:**
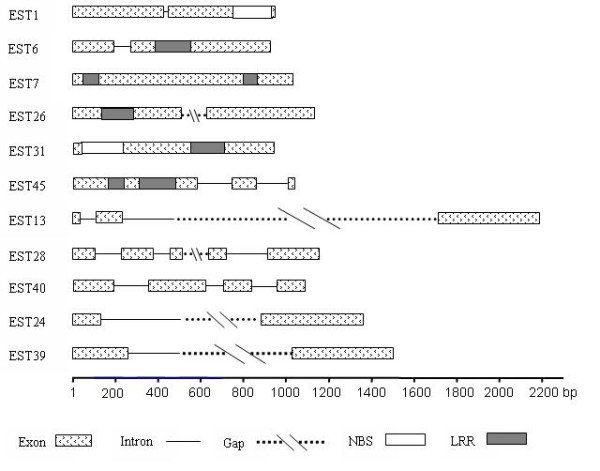
Gene structures of 11 candidate resistance genes.

11 primer pairs were used to amplify 11 candidate gene fragments with sizes of 932 to 2200 bp (EU054285 - EU054395 Table 1 and Fig. 1). The 11 genes included 6 NBS-LRR like genes, 2 PKpA genes, 1 MAPK gene, 1 EDR, and 1 PR gene. The sequenced fragments of three candidate genes (EST7, EST26, and EST31) contained exclusively coding regions. All other genes included intron sequences in addition. A total of 10,971 bp were aligned over all loci for the 20 genotypes, the length of sequence alignment for each gene was about 1 kb (904–1085 bp), which is used to develop markers for candidate gene allele tracing in selection experiments (unpublished results).

**Table 1:** Summary information on allele sequences for 11 candidate genes obtained from the 20 diploid heterozygous *L. perenne *genotypes within the LTS

Genes	Homologues	Fragment length (bp)^a^	Sequenced (bp)^b^	No. of haplotypes	Primers for gene fragments (5' to 3')
EST1	NBS-LRR	949	949	27	F ctcggatccaacatggagga R tgttgtcttgccaataccgc
EST6	NBS-LRR	936	932	27	F gggaaagtccaaacttgacg R cgtaagaatgggtgaaaggt
EST7	NBS-LRR	1036	1036	19	F atagccttcctttggcaatc R ctggacaacgagttacacgg R
EST26	NBS-LRR	1140	507 + 523	14	F gctgcagctggctaacaaca R ccaaatgtgccagcaactgc
EST31	NBS-LRR	943	943	12	F agcacgccatcactgttcta R ctagggcatcaaccgactgt
EST45	NBS-LRR	1014	1056	23	F gagcagccttcctccaaact R caaggccacgagaactagca
EST13	EDR-1	2200	480 + 479	10	F aagcggaggattaagatggc R cacatattcacatgggacgc
EST24	MAP-1	1350	505 + 480	13	F tatatccgccaacttccccg R tcaatcatcacctgcccacc
EST28	PKPA	1145	509 + 510	10	F gagcaacaagactgaccatt R caatctggtttgttcttggc
EST39	PKPA	1500	502 + 475	15	F cacatcatcggattccacaa R atacatcccaatccacctgg
EST40	PRP-1	1085	1085	9	F agaaacaggaggcgacaagt R ggagtgatcgtccttttaca

^a ^Fragment length responds to largest PCR band among 20 genotypes.

^b ^Length of fragments sequenced for all 20 LTS genotypes.

The number of haplotypes among the 11 genes in the 20 heterozygous genotypes ranged from 9 in EST40 to 27 in both EST1 and EST6, with an average of 16.3 alleles per gene. On average, 20.4 and 11.4 alleles were detected for NBS-LRR and Non-NBS-LRR genes, respectively (Table 2). 140 alleles (78%) appeared at low frequency of less than 5% over the 11 genes (i.e., these alleles were present in max. 2 copies within the LTS genotypes). Only eight alleles showed high frequencies of more than 20%. Two alleles for EST26 and EST28 were present at high frequencies (45.0 and 52.5%, respectively).

**Table 2:** Allele frequencies and homozygosity for 11 genes based on the 20 LTS genotypes

		Allele frequencies				Homozygosity %	
Genes	Alleles	≤5%	>5–20%	>20–40%	>40–60%	Observed	Expected

EST1	27	24	3	0	0	20.0**	4.5
EST6	27	24	3	0	0	20.0**	4.2
EST7	19	13	6	0	0	40.0**	5.4
EST26	14	10	3	0	1	55.0**	14.9
EST31	12	9	2	1	0	55.0**	18.6
EST45	23	20	3	0	0	60.0**	7.9
N-L average^a^	20.4	16.6	3.3	0.3	0.3		
EST13	10	6	3	1	0	45.0**	22.8
EST24	13	9	3	0	1	35.0*	22.3
EST28	10	7	2	0	1	55.0**	33.4
EST39	15	13	1	1	0	45.0**	17.2
EST40	9	5	2	2	0	75.0**	26.0
R average^b^	11.4	8.0	2.2	0.8	0.4		
Average^c^	16.3	12.7	2.8	0.5	0.3	45.9**	16.1

*, ** significant differences between observed and expected homozygosity at the level of 5% and 1%, respectively, by Chi square test.

^a ^N-L average: average across the 6 NBS-LRR like genes.

^b ^R average: average across the other 5 R genes.

^c ^Average: average across all the 11 genes.

Based on marker haplotypes, the homozygosity per candidate gene within the LTS genotype collection ranged from 20 to 75%, which significantly exceeded the percentages expected in a single panmictic ryegrass super-population (Table 2). Across the 11 genes, the 20 LTS genotypes were 45.9% homozygous, ranging from 29% in LTS19 to 85% in LTS02 (Table 3).

**Table 3:** Description of diploid heterozygous *L. perenne *genotypes within the Lolium Test Set (LTS)

Code	Name	Type	Specificity	Country^a^	Homozygosity (%)^b^
LTS01	G00612	Forage	Parent in a mapping population	NL	36
LTS02	G00559	Forage	Parent in a mapping population	NL	85
LTS03	NGB9C2	Ecotype	Parent in a mapping population	DK	54
LTS04	Veyo9C1	Forage	Parent in a mapping population	DK	50
LTS05	DLF5	Turf	Parent in a mapping population	DK	36
LTS06	DLF6	Turf	Parent in a mapping population	DK	64
LTS07	G00851	Forage	Parent in a mapping population	NL	43
LTS08	G00852	Forage	Parent in a mapping population	NL	57
LTS09	RASP17-03	Forage	RASP family self-fertility S_1F_S5_1F_	UK	64
LTS10	ILGI 80	Forage	Selected genotype from ILGI mapping population	UK	38
LTS11	Lp 34–551	Turf	Colchicine induced type	LT	57
LTS12	INRA1	Forage	Parent in a mapping population	F	64
LTS13	INRA2	Forage	Parent in a mapping population	F	38
LTS14	INRA3	Turf	Parent in a mapping population	F	57
LTS15	INRA4	Ecotype	Mediterranean origin: Greece	F	43
LTS16	INRA5	Ecotype	Nordic origin: Sweden	F	38
LTS17	WSC 22/9	Forage	Selected genotype from WSC mapping population	UK	48
LTS18	WSC 23/9	Forage	Selected genotype from WSC mapping population	UK	36
LTS19	ILGI P150/112 74	Forage	Selected genotype from ILGI mapping population	UK	29
LTS20	ILGI P150/112 166	Forage	Selected genotype from ILGI mapping population	UK	50

^a ^NL, The Netherlands; DK, Denmark; UK, United Kingdom; LT, Lithuania; F, France.

^b ^Percentage of homozygous loci among all the 11 genes based on the sequenced 1 kb allele sequences.

### SNP and Indel polymorphisms

The aligned 10,971 bp included 332 insertion-deletion mutations (Table 4). Indels were observed in nine gene fragments except for EST26 and EST31. For one out of eight genes spanning both coding and non-coding regions, Indels were only observed in non-coding regions, whereas for three genes, Indels were only observed in coding regions. For the remaining four genes, Indels were observed both in the coding and non-coding regions, and their frequency in the non-coding region was substantially higher than in the coding region.

**Table 4:** Summary of DNA polymorphism and diversity estimates in the about 1000 bp of 11 candidate genes

Parameters	Entire region	Non-coding	Coding regions		
			
			All sites	Synonymous	Non-synonymous
**EST1 **(bp)	949	33	916		
Indels (sites)	57	12	45		
SNP sites	107	0	107	25	82
Polymorphic sites in %^a^	12.0	0	12.3	12.0	12.6
θ/bp^b^	0.0297	0	0.0301		
Tajima's D^b^	-0.0676^ns^	-	-0.0676^ns^		
**EST6 **(bp)	936	95	841		
Indels (sites)	67	0	67		
SNP sites	247	17	230	71^c^	137^c^
Polymorphic sites in %	28.4	17.9	29.7	40.3	22.0
θ/bp	0.0793	0.0553	0.0829		
Tajima's D	-0.4662^ns^	0.0302^ns^	-0.5024^ns^		
**EST7 **(bp)	1036	0	1036		
Indels (sites)	13		13		
SNP sites	277		277	57^c^	183^c^
Polymorphic sites in %	27.1		27.1	27.1	22.9
θ/bp	0.0791		0.0791		
Tajima's D	-0.3672^ns^		-0.3672^ns^		
**EST26 **(bp)	1030	0	1030		
Indels (sites)	0		0		
SNP sites	16		16	6	10
Polymorphic sites in %	1.6		1.6	2.6	1.3
θ/bp	0.00365		0.00365	0.0060	0.0030
Tajima's D	0.0986^ns^		0.0986^ns^		
**EST31 **(bp)	943	0	943		
Indels (sites)	0		0		
SNP sites	20		20	0	20
Polymorphic sites in %	2.1		2.1	0	2.7
θ/bp	0.0050		0.0050	0	0.0064
Tajima's D	1.1385^ns^		1.1385^ns^		
**EST45 **(bp)	1056	309	747		
Indels (sites)	87	0	87		
SNP sites	258	91	167	32^c^	107^c^
Polymorphic sites in %	26.6	29.4	22.4	21.8	21.7
θ/bp	0.0756	0.0896	0.0691		
Tajima's D	-0.4209^ns^	-0.3269^ns^	-0.4715^ns^		
**EST13 **(bp)	959	326	633		
Indels (sites)	9	5	4		
SNP sites	9	2	7	3	4
Polymorphic sites in %	0.9	6.2	1.1	2.2	0.8
θ/bp	0.0022	0.0015	0.0026	0.0051	0.0020
Tajima's D	0.8068^ns^	1.0927^ns^	0.5250^ns^		
**EST24 **(bp)	985	385	600		
Indels (sites)	11	7	4		
SNP sites	37	25	12	8	4
Polymorphic sites in %	3.8	6.6	2.0	6.1	0.9
θ/bp	0.0089	0.0160	0.0047	0.0144	0.0020
Tajima's D	1.1897^ns^	1.1556^ns^	1.0406^ns^		
**EST28 **(bp)	1019	398	621		
Indels (sites)	58	58	0		
SNP sites	63	33	30	20	10
Polymorphic sites in %	6.6	9.7	4.8	14.8	2.1
θ/bp	0.0154	0.0228	0.0114	0.0347	0.0049
Tajima's D	0.8363^ns^	1.3632^ns^	0.1900^ns^		
**EST39 **(bp)	977	257	720		
Indels (sites)	28	23	5		
SNP sites	52	13	39	24^c^	10^c^
Polymorphic sites in %	5.5	5.4	5.5	12.8	1.9
θ/bp	0.0130	0.0138	0.0138		
Tajima's D	2.1562*	0.9752^ns^	2.4360*		
**EST40 **(bp)	1085	371	712		
Indels (sites)	2	0	2		
SNP sites	9	2	7	1	6
Polymorphic sites in %	0.8	0.5	1.0	0.6	1.1
θ/bp	0.0020	0.0013	0.0023	0.0013	0.0027
Tajima's D	-0.1300^ns^	1.2987^ns^	-0.7024^ns^		

^a ^Polymorphic sites in percentage measured as polymorphic sites in the target region divided by the total nucleotides in the region excluding indels. Synonymous (non-synonymous) polymorphic sites in percentage measured as synonymous (non-synonymous) mutation sites divided by synonymous (non-synonymous) sites.

^b ^θ Watterson's estimator; π nucleotide diversity per site; D Tajimas's D: *, ** significant at P = 0.05 and 0.01 level; ns non-significant.

^c ^A number of synonymous and non-synonymous mutations were not included due to some codons with multiple and complex evolutionary path.

Excluding Indels, the length of aligned sequences was 10,658 bp. There were 1095 SNPs in the 10,658 bp (Tables 4 and 5), which is 1 SNP per 10 bp within the LTS, representing 40 alleles per locus. Out of those, 135 sites were tri-allelic, and only 19 sites were tetra-allelic. Among the 11 genes, the number of SNPs varied substantially from 9 in EST40 to 277 in EST7 within about 1000 bp (Table 4). Three gene fragments (EST6, EST7, and EST45) showed a high percentage of SNP polymorphisms (>25%). In contrast, only 0.8% polymorphic sites were detected in the 1 kb region of EST40. Candidate genes with a high density of SNPs such as EST1, EST6, EST7, and EST45 showed singletons for many sites, as well as the majority of sites with 3 or 4 SNP variants.

**Table 5:** Comparison of nucleotide diversity in different gene classes for the 20 LTS genotypes

Genes^a^	SNP	θ^b^	π^c^	D^d^
N-L	925	0.0464	0.0427	-0.2998
R	170	0.0089	0.0062	1.5486
All genes	1095	0.0306	0.0314	0.0203

^a ^NBS, R, and All genes means the merged sequence of NBS-LRR genes, non-NBS-LRR genes, and all the 11 genes, respectively, when calculation.

^b ^θ Watterson's estimator;^c ^π nucleotide diversity per site;^d ^D Tajimas's D

On average, the percentage of polymorphic sites in non-coding regions was two-fold higher than in coding regions of EST13, EST24, and EST28. For three genes (EST39, EST40 and EST45), there was a similar SNP density in non-coding and coding regions. Two genes displayed a higher SNP density in coding compared to non-coding regions: EST1 and EST6. For three gene fragments containing exclusively coding regions, the SNP density varied substantially with 277 SNPs in EST7, and only 16 and 20 SNPs in about 1000 bp of EST26 and EST31, respectively.

Across all 11 genes, 1 SNP every 33 bp (θ/bp = 0.0306, Table 5) was found between two randomly sampled sequences. However, the SNP density differed substantially between gene classes. NBS-LRR genes showed a very high SNP density of one SNP every 22 bp between two randomly sampled sequences, whereas non-NBS-LRR genes showed a limited SNP density of one SNP every 112 bp.

### Nucleotide diversity

Three NBS-LRR genes, EST6, EST7, and EST45, showed the highest pairwise nucleotide diversities (π > 0.06) among the 20 LTS genotypes (Table 4), whereas EST13 and EST40 showed the lowest pairwise nucleotide diversities (π < 0.003). For four out of the eight candidates with sequences from both coding and non-coding regions, the coding regions showed higher pairwise nucleotide diversities than the corresponding non-coding regions. The synonymous mutation rate was about two-fold higher than the non-synonymous mutation rate for EST6, EST13, EST24, EST26, EST28, and EST39. The non-synonymous mutation rates for EST31 and EST40 were about 2-times higher than synonymous mutation rates. For the remaining three genes, synonymous and non-synonymous mutations were present at a similar frequencies (not significant at p = 0.05).

### Selection

Tajima's D was negative and not significant for four candidate genes, indicating that a few alleles predominated, whereas most other alleles showed low frequencies (Tables 2 and 4). For the remaining seven genes, positive Tajima's D values were obtained from the 20 LTS genotypes. Tajima's D statistic for EST39 was significant for the 20 LTS genotypes at the level of p = 0.05, for both coding and the entire 1 kb region.

### LD decay

**Figure 2:**
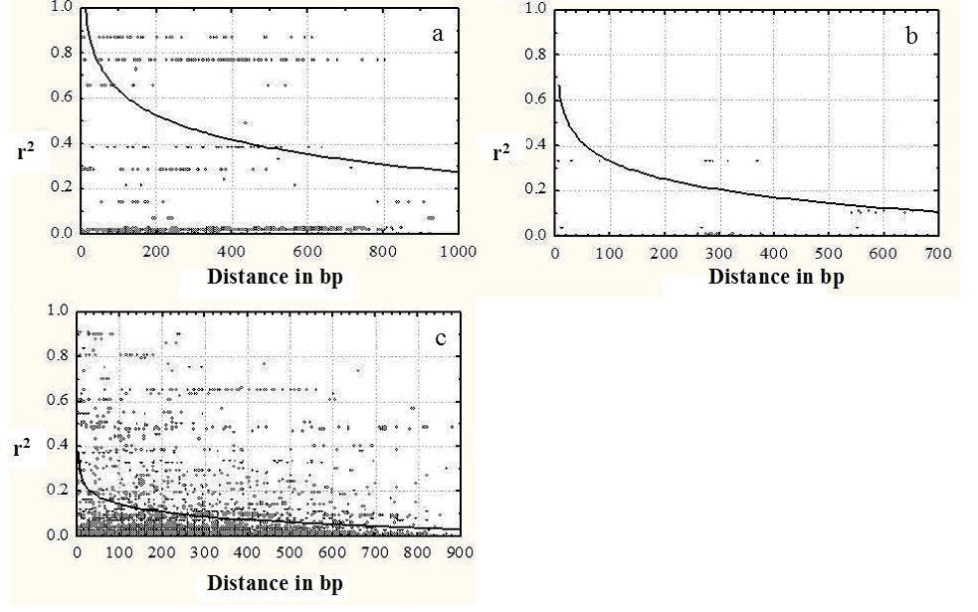
Plots of squared correlations of allele frequencies (r^2^) against distance between pairs of polymorphic sites in three genes: a) EST28, b) EST13, and c) EST1. Curves show nonlinear regression of r^2 ^on weighted distance.

For all studied NBS-LRR genes, except for EST31, LD decayed within 15–25 bp (Table 6). In contrast, LD decayed within 300–900 bp for the Non-NBS-LRR genes (Figure 2b). A higher level of LD exceeding the sequenced 1 kb region was found for EST 28 (Figure 2a). Very low LD was detected for EST1 (Figure 2c), EST6, EST7, EST26, and EST45 (average r^2 ^< 0.12, < 15% of pairwise comparisons significant at 0.01 level) (Table 6). Out of those, only EST26 contained a small number of SNPs (16) and showed a low degree of nucleotide diversity (θ = 0.0037). For the other four genes, a large number of SNPs (more than 100) were detected in the sequenced 1 kb region. Seven genes showed low levels of LD with r^2 ^values below 0.2 within distances of 400 bp (average r^2 ^> 0.17, > 21% of pairwise comparisons significant at 0.01) (Table 6, Figure 2).

**Table 6:** Intragenic LD values between pairs of polymorphic sites and numbers of site pairs showing LD at P = 0.01 level within one gene

	r^2 ^Mean ± SD	Distance r^2 ^< 0.2^a^	D' Mean ± SD	No of pairwise comparisons	
				
Genes				In LD^b^	Totally tested
EST1	0.11 ± 0.22	25	-0.3929 ± 0.8585	696 (13.5)	5151
EST6	0.11 ± 0.23	20	-0.4139 ± 0.8443	2032 (9.9)	20503
EST7	0.10 ± 0.22	20	-0.5966 ± 0.7678	2251 (9.2)	24531
EST26	0.11 ± 0.14	25	-0.4264 ± 0.8885	15 (12.4)	120
EST31	0.19 ± 0.26	220	-0.1701 ± 0.8141	63 (33.2)	190
EST45	0.07 ± 0.17	15	-56536 ± 0.7635	1666 (7.9)	21115
EST13	0.28 ± 0.35	300	0.02156 ± 0.9371	15 (41.7)	36
EST24	0.23 ± 0.30	500	-0.4334 ± 0.8558	223 (33.5)	666
EST28	0.54 ± 0.46	900 (1.6)	0.1887 ± 0.9804	1128 (57.8)	1953
EST39	0.29 ± 0.31	710	-0.2859 ± 0.8581	635 (54.0)	1176
EST40	0.21 ± 0.32	500	0.1158 ± 0.9085	11 (30.6)	36

r^2 ^= ZnS (Kelly 1997), average of r^2 ^over all pairwise comparisons; D' (Lewontin 1964)

^a ^Distance in bp, but the numbers in bracket were calculated based on the function between distance and r^2 ^in kb.

^b ^The significant association between polymorphic pairs determined by the two-tailed Fisher's exact test. Number in bracket means the percentage, which significant pairs accounted of total pairwise comparisons.

## Discussion and conclusion

### Variable nucleotide diversity among 11 expressed resistance candidate genes

The findings of this study are in agreement with high levels of genetic diversity within the out-crossing species *Lolium perenne*. The pairwise nucleotide diversity for our sample of genes and genotypes of one SNP every 33 bp (π = 0.0314) (or 1 SNP per 10 bp across all 20 LTS genotypes) was higher than in several other studies [21-26], where pairwise nucleotide densities ranged from 1 SNP per 60 bp (π = 0.0171) in a 20-kb interval containing the *Arabidopsis thaliana *disease resistance gene *RPS5 *[17], to 1 SNP per 1030 bp in soybean [26]. SNP densities varied substantially between ryegrass genes, ranging from 1 SNP per 13 bp in three NBS-LRR genes (EST6, EST7, and EST45) to 1 SNP per 500 bp in a *PkpA *gene (EST40). The overall high SNP density was mainly caused by the three genes EST6, EST7, and EST45, with more than 200 SNP sites within 1 kb. When excluding these three genes, the average SNP density decreased to 1 SNP per 26 bp in our sample of 20 LTS genotypes, which was similar to the SNP density of 1 SNP per 28 bp detected on maize chromosome 1 for 25 genotypes [14] and 1 SNP per 26 bp in 22 accessions of *Arabidopsis thaliana *[17]. Due to the organisation of NBS-LRR genes in large gene families, amplification and sequencing of paralogues rather than allelic sequences might have lead to the high SNP densities for these three genes. However, there was neither a sub-grouping of "allele sequences" within these genes (indicative of sequences from at least two different genes), nor single very different "alleles", which lead to the high SNP densities. After removing the most divergent alleles for these three genes, the total number of SNPs did not decrease substantially and still was above 200 per gene. Therefore, alleles within these genes seem to be highly variable, which might be in agreement with an active role in multiallelic gene-by-gene interactions with pathogen isolates (all of them belong to the NBS-LRR group). This is further supported by the finding, that the maximum number of haplotypes per gene, 20.4, was identified among the NBS-LRR gene class, whereas a substantially lower number of haplotypes, 11.4, was found for non-NBS-LRR resistance gene candidates.

However, high SNP densities were only detected for some but not all NBS-LRR genes. Possibly NBS-LRR genes with limited allele variability interact with pathogens with only low numbers of pathotypes (like some viruses), or are of an evolutionary recent origin. Another reason for the large differences in SNP densities between NBS-LRR genes might be that the sequenced 1 kb regions were located in different parts of the genes, which might contain conserved regions (like NB domain) or hypervariable regions such as the solvent-exposed positions of the LRRs [27,28]. For example, the sequenced 1 kb region of EST6, 7, and 45 with high SNP densities included hypervariable regions.

### High homozygosity

The observed heterozygosity of the 20 LTS genotypes determined by SNP haplotypes was 2-times lower than expected. Since only five PCR fragments were sequenced per genotype, some alleles might have escaped for statistical reasons, or due to preferential amplification of one out of two alleles within a heterozygous genotype. However, these reasons cannot explain for the large discrepancy between observed and expected heterozygosity. Another explanation is, that the 20 genotypes collected from different regions in Europe suffered from regional isolation, with only a limited number of alleles segregating in each of the regions. The most likely explanation is that several of the LTS genotypes originate from breeding programs, with some degree of inbreeding.

### Natural selection resulting in high levels of sequence diversity within R genes

In theory, silent mutations including mutations in noncoding regions and synonymous mutations in coding regions have less severe phenotypic effects than non-synonymous mutations, changing the amino-acid composition. Thus, a relatively higher proportion of silent mutations are expected for "functional genes" underlying natural selection. However, in this study, only three (EST13, EST24, and EST 28) out of 8 genes showed 2-fold more polymorphic sites in noncoding regions than in coding regions. Significantly higher polymorphism rates in coding than in noncoding regions were detected in EST1 and EST6. For the other three genes, the frequency of segregating sites was similar in both coding and noncoding regions. R genes showed very high levels of nucleotide diversity in other studies [29,30]. High frequencies of polymorphic sites in coding regions, ranging from 12.3% to 29.7%, were observed in the four NBS-LRR genes EST1, EST6, EST7, and EST45. Probably no or little selection pressure occurred at these loci during evolution, so that several mutations could be maintained. In addition, these genes were identified as cDNA sequences, and should therefore, not be pseudogenes. However, in some cases alleles might have turned into non-expressed "pseudo-alleles", which might mutate more rapidly.

The LRR domain of R proteins of plants is suggested to interact directly or indirectly with pathogen elicitors to determine race specificity. Hypervariability in the lettuce *RGC2 *family involved in pathogen recognition was observed in the 3'-encoded LRR domain. Moreover, two times higher rates of nonsynonymous than synonymous substitutions were detected [27]. The study of the complete NBS-LRR gene family in the Arabidopsis genome showed that LRRs were hypervariable and subject to positive natural selection, approximately 70% of the positively selected sites are located in the LRR domain, whereas the remaining 30% are located outside the LRR domain [19]. In this study, four NBS-LRR like gene fragments (EST1, EST6, EST7, and EST45), each with about 20 haplotypes, showed very high nonsynonymous mutation rates (12.6–22.9%), leading to altered amino acid sequences. Three of them included LRR regions. For EST31, only nonsynonymous mutations were found, indicative of positive selection. Particularly LRR regions showed very high diversity with one SNP every 10 bp between two sequences (θ = 0.10) on average. However, this high nonsynonymous rate was only observed for NBS-LRR genes but not for the other genes investigated in this study. This is in agreement with a study of sequence diversity of 27 R genes in Arabidopsis [31].

Distinct forms of selection produce specific patterns of sequence diversity [32]. Plant – pathogen interactions tend to increase the amount of genomic mutations in R genes in the long process of natural selection. Neutral theory of molecular evolution [33] classified mutations into three types: neutral (unchanged function), deleterious (eliminated by selection), and beneficial (too rare to be noticed). According to neutral evolution theory, silent mutations should be randomly maintained in the long history of evolution. Therefore, only neutral variation should be observed. In this study, 10 out of 11 genes followed the 0-hypothesis (neutrality). The only exception was EST39: Tajima's D statistics was significant for EST39 among the 20 LTS, indicating that the neutral mutation hypothesis cannot explain the occurrence of the mutations both in the coding and the entire 1 kb region. However, the disease resistance system in plants seems to preserve rare alleles, since 78% of alleles for the 11 genes were rare alleles (81.4% for NBS-LRR and 70.2% for non-NBS-LRR). Strong natural selection pressures are expected on genes involved in recognition mechanisms in host-pathogen relationships [34]. Therefore, fast evolutionary patterns should result from the competition between infection and defence systems, and increase allelic diversity. On the other hand, disease resistance is a very important fitness trait, thus high polymorphism in R genes may be the consequence of natural selection that maintain both resistance and susceptibility alleles. There might in addition be different pathogen virulences present in different regions of the world, leading to maintenance of different resistance alleles in distinct regions. However, also absence of selection pressure (neutral mutations) could explain for large variation within genes. Thus evolution creates an excess of "silent" R genes, which "wait" for novel pathogen virulence genes in future.

### LD association mapping for QTL

Population mating patterns and admixture can influence LD. Generally, LD decays more rapidly in outcrossing species as compared to selfing species [35]. When the rate of LD decay is rapid, LD mapping is potentially very precise. The factors affecting the number of sensory hairs were mapped by LD mapping on *Drosophila thorax *[36]. In maize, rapid LD decay at the *d8 *locus was prerequisite to detect associations of polymorphisms between SNP and INDEL polymorphisms in the *d8 *gene with plant height and flowering time [16]. Skøt et al. [15] conducted association mapping to identify flowering time genes using AFLP markers in natural populations of *Lolium perenne*. They found three closely linked markers within a major QTL region on chromosome 7 highly associated with heading date. They suggested that association mapping approaches maybe feasible at the marker level in *L. perenne*. However, the majority of all pairwise comparisons did not show significant LD at the level of p = 0.05. If the threshold of significant LD value was set to 0.2, there was no LD among linked marker pairs in their study, which is in agreement with the low LD found in our study. Noel et al. [37] calculated LD statistics for drought tolerance-associated *Lp*ASRa2 SNPs using 35 diverse perennial ryegrass individuals. They found very limited intragenic LD. In this study, substantial LD decay was found within a physical distance of 500 bp for most genes. Thus for a whole genome scan, either a very dense marker coverage (1 marker each few hundred bp) or experimental populations with higher LD would be required. However, for candidate gene based association studies, a very high genetic resolution can be expected, when working with natural populations in *L. perenne*. Hence, LD based association analysis is feasible within candidate genes and promising for QTL fine mapping in ryegrass.

## Methods

### Plant materials

A total of 20 genotypes of perennial ryegrass (*Lolium perenne *L.) originating from various European sources (Table 1) were included in this study. They were classified into three subgroups: forage with 13, ecotype with 3, and turf with 4 genotypes. These genotypes represent a wide range of genetic diversity within ryegrass [38].

### Allele sequencing of candidate genes

11 potential disease resistance genes were selected from the annotation of EST sequences generated within the Danish genome project DAFGRI [39], which included homologues of nucleotide binding site and leucine rich repeat (NBS-LRR), pathogenesis response (PR), Mitogen-activated protein kinase (MAPK), enhanced disease resistance (EDR), and plastid pyruvate kinase A (PKpA) protein coding genes (Table 2). On the basis of candidate mRNA sequences, 11 pairs of primers were designed to amplify about 1 kb genomic fragments from the 20 genotypes for each of the 11 genes (Table 2). A touch down PCR program was used beginning with 5 min at 94°C, followed by 12 cycles of 30 s at 94°C, 60 s at annealing temperature 67°C, 60 s at 72°C with the annealing temperature decreasing by 1°C per cycle, followed by 29 cycles of 30 s at 94°C, 60 s at 55°C, 60 s at 72°C and 10 min at 72°C. All 11 primer pairs ran with the same PCR program on a MJ Research thermocycler (Applied Biosystems, Califonia) in 25 μl reaction mixtures containing 20 ng DNA, 0.2 μM primer, 0.2 mM dNTPs, 0.4 u BD Advantage 2 polymerase (BD Biosciences Clontech, Palo Alto, CA), and 2.5 μl 10 × BD advantage 2 PCR buffer.

PCR products were purified from agarose gel using QiaQuick spin columns (Qiagen, Valencia, USA) according to manufacturer instructions. Purified fragments were cloned into vector pCR^®^2.1-TOPO (TOPO TA cloning kit, Invitrogen, Califonia). Five clones per gene for each genotype were randomly picked for plasmid DNA extraction. Purified plasmid DNA was used for allele sequencing on the MegaBACE1000 (Amersham Bioscience, Califonia). Sequence chromatogram files from the same genotype were assembled into contigs by using SEQMAN (DNA star, Madison, WI), and consensus sequences were edited manually to resolve discrepancies. Consensus sequences across all 20 genotypes were aligned by using CLUSTAL. Polymorphisms which appeared only in one genotype were manually rechecked in chromatogram files.

### Data analysis

When calculating the number of haplotypes, all polypmorphic sites including Indels and segregating sites with two and more variants were taken into consideration. Direct comparison of mRNA sequence and its corresponding genomic DNA sequences was used to determine exon and intron regions. All calculations were based on 40 alleles from the 20 heterozygous diploid genotypes. If one genotype was homozygous in the sequenced region, its allele sequence was presented twice in the alignment in order to determine the allele frequency for the 20 genotypes. Alignment data for each candidate gene were used for nucleotide diversity and linkage disequilibrium (LD) analysis. DnaSP version 4 [40] was used for the following analyses.

Nucleotide diversity was evaluated by the parameter π, which is the average number of nucleotide differences per site between two sequences [41]. The neutral mutation parameter θ was calculated from the total number of mutations [42]. Tajima's D statistic [43] was used to test for neutral selection. LD was estimated by using standardized disequilibrium coefficients (D') [8] and squared allele-frequency correlations (r^2^) [9] for pairs of SNP loci. Sites with alignment gaps or polymorphic sites segregating for three or four nucleotides were completely excluded from the analysis. Fisher's exact test [44] was used to determine the statistical significance of LD. Decay of LD with distance in base pairs (bp) between sites within the same gene was evaluated by nonlinear regression in Statistica [45].

## List of abbreviations

LTS lolium test set; SNP single nucleotide polymorphism; LD linkage disequilibrium; NBS-LRR nucleotide binding site and leucine rich repeat.

## Authors' contributions

YX cloned the candidate R genes and carried out allele sequencing. UF along with YX did the data analysis. BS along with YX chose the R genes and designed primers for 1 kb fragments. TA provided EST sequence information. TL coordinated the project and along with YX wrote the manuscript. All the authors read and approved the final manuscript.
